# CSF amino acid profiles in ICV‐streptozotocin‐induced sporadic Alzheimer's disease in male Wistar rat: a metabolomics and systems biology perspective

**DOI:** 10.1002/2211-5463.13814

**Published:** 2024-05-20

**Authors:** Amir Barzegar Behrooz, Hamid Latifi‐Navid, Jabar Lotfi, Fariba Khodagholi, Shahla Shojaei, Saeid Ghavami, Javad Fahanik Babaei

**Affiliations:** ^1^ Electrophysiology Research Center, Neuroscience Institute Tehran University of Medical Sciences Iran; ^2^ Department of Human Anatomy and Cell Science, College of Medicine University of Manitoba Winnipeg Canada; ^3^ Department of Molecular Medicine National Institute of Genetic Engineering and Biotechnology Tehran Iran; ^4^ School of Biological Sciences Institute for Research in Fundamental Sciences (IPM) Tehran Iran; ^5^ Growth and Development Research Center Tehran University of Medical Sciences Iran; ^6^ Neuroscience Research Center Shahid Beheshti University of Medical Sciences Tehran Iran; ^7^ Faculty of Medicine in Zabrze University of Technology in Katowice Zabrze Poland; ^8^ Research Institute of Oncology and Hematology Cancer Care Manitoba‐University of Manitoba Winnipeg Canada; ^9^ Children Hospital Research Institute of Manitoba University of Manitoba Winnipeg Canada

**Keywords:** glucose metabolism, neurometabolic, sporadic Alzheimer's Disease, streptozotocin, systems biology

## Abstract

Alzheimer's disease (AD) is an increasingly important public health concern due to the increasing proportion of older individuals within the general population. The impairment of processes responsible for adequate brain energy supply primarily determines the early features of the aging process. Restricting brain energy supply results in brain hypometabolism prior to clinical symptoms and is anatomically and functionally associated with cognitive impairment. The present study investigated changes in metabolic profiles induced by intracerebroventricular‐streptozotocin (ICV‐STZ) in an AD‐like animal model. To this end, male Wistar rats received a single injection of STZ (3 mg·kg^−1^) by ICV (2.5 μL into each ventricle for 5 min on each side). In the second week after receiving ICV‐STZ, rats were tested for cognitive performance using the Morris Water Maze test and subsequently prepared for positron emission tomography (PET) to confirm AD‐like symptoms. Tandem Mass Spectrometry (MS/MS) analysis was used to detect amino acid changes in cerebrospinal fluid (CFS) samples. Our metabolomics study revealed a reduction in the concentrations of various amino acids (alanine, arginine, aspartic acid, glutamic acid, glycine, isoleucine, methionine, phenylalanine, proline, serine, threonine, tryptophane, tyrosine, and valine) in CSF of ICV‐STZ‐treated animals as compared to controls rats. The results of the current study indicate amino acid levels could potentially be considered targets of nutritional and/or pharmacological interventions to interfere with AD progression.

## Graphical abstract

A low dose of streptozotocin (STZ) intracerebroventricularly (ICV) into the brain was used to reproduce Alzheimer's disease (AD) pathogenesis. Amino acid profiling of cerebrospinal fluid (CSF) was investigated to uncover potential disease biomarkers. Our results showed a reduction in the concentrations of various amino acids upon ICV‐STZ administration which could be potential targets to interfere with AD progression.

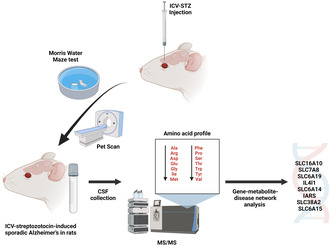

**Abbreviations**
AAs: aromatic amino acids
ACMS: α‐amino‐β‐carboxymuconate‐ε‐semialdehyde
AD: Alzheimer's disease
Aβ: amyloid plaques
BBR: brain‐to‐background ratio
BCAAs: branch‐chain amino acids
FDR: false discovery rate
GABA: gamma‐aminobutyric acid
HMDB: human metabolome database
ICV‐STZ: intracerebroventricular‐streptozotocin
KEGG: Kyoto Encyclopedia of Genes and Genomes
KP: kynurenine pathway
MRM: multiple reaction monitoring
MS: mass spectrometry
MWM: Morris Water Maze test
NFTs: neurofibrillary tangles
NGF: neural growth factor
PD: Parkinson's disease
PET: positron emission tomography
sAD: sporadic Alzheimer's disease
SLCs: solute carriers
STZ: streptozotocin

The number of people living with dementia worldwide currently exceeds 50 million worldwide and is increasing rapidly each year [1]. Alzheimer's disease (AD) is a progressive neurodegenerative disease and the most common cause of dementia, resulting in loss of memory and other cognitive abilities. Increasing evidence suggests that the pathology of AD is complex and extends beyond its typical pathological characteristics, such as amyloid plaques (Aβ) and neurofibrillary tangles (NFTs) [2]. Indeed, the disease is also characterized by systemic abnormalities and metabolic aberrations of the brain, which are evident at the molecular and biochemical levels [3].

It has been indicated that around 95% of individuals diagnosed with Alzheimer's disease suffer from the sporadic form known as sAD. This form of disease is influenced by multiple factors, including genetics, metabolism, and environmental risks [4, 5]. In animal studies, administering low doses of streptozotocin (STZ) intracerebroventricularly (ICV) into the brain has been shown to interfere with brain insulin signaling, leading to impaired cerebral glucose metabolism. This disruption can cause neuropathological and biochemical changes similar to those observed in the pathology of sAD. Indeed, multiple studies have demonstrated that ICV administration of STZ to rodents reproduced many features of AD pathogenesis at the cellular level, particularly in the cortex and hippocampus; this led to memory and behavioral deficits, with changes occurring gradually and persisting for an extended period [6, 7, 8, 9]. Thus, STZ ICV administration is a valuable tool for studying the mechanisms underlying sAD, potentially providing insights to develop new treatments [10, 11], and represents a good preclinical model for testing drugs targeting specific pathways, such as insulin signaling [12]. The molecular signaling pathway in the STZ‐induced sAD model is not yet fully understood.

Considering the observed deficit in glucose metabolism in AD brain neurons, exploring alternative energy sources could represent a potential solution to prevent neuronal death associated with AD. Neurons may rely on other energy sources, such as amino acids, ketone bodies, citric acid cycle intermediates, pyruvate, and lactate, as they lack the necessary enzymes for the beta‐oxidation of fatty acids [13]. Recent metabolomic studies have revealed alterations in amino acid levels in the brain tissue, plasma, and cerebrospinal fluid of Alzheimer's patients, suggesting these metabolic changes may play a role in disease development and progression. Therefore, supplementing or replenishing the brain with these essential factors and metabolites could represent a promising approach to effectively combat AD [13, 14].

Alzheimer's disease is a complex multifactorial disease involving genomic, interactome, and environmental factors, intrinsic genomic susceptibility, and constant dynamic interaction between impaired pathways and central homeostatic nerve cell networks. New holistic systems‐level experimental and computational methods are needed to study AD's complexity. Systems biology approaches may reveal fundamental insights, processes, networks, and interactions [15]. Metabolomics and biomarkers used for AD screening may assist in improving early disease diagnosis and treatment approaches from a systems biology viewpoint, considering metabolic changes might occur before clinical indications appear [16]. Metabolomics‐based MS/MS techniques have been proposed to identify (new) AD diagnostic biomarkers such as amino acids [17, 18, 19, 20, 21]. As neurotransmitters, metabolic regulators, and neuromodulators, amino acids (AAs) fulfill vital central nervous system functions (CNS) functions. As such, profiling of amino acids could be regarded as a method for identifying diagnostic biomarkers of neurodegenerative diseases like AD [22, 23]. In AD, evidence indicates that metabolic network failures can provide a biochemical roadmap. For instance, a multivariate‐adjusted analysis showed that sphingomyelins and ether‐containing phosphatidylcholines were altered in preclinical biomarker‐defined AD stages, while acylcarnitines and various amines, including valine and α‐aminoadipic acid, changed in the symptomatic state [24]. In another study, researchers identified significant changes in the metabolite profile of AD patients compared to healthy controls. Furthermore, higher levels of cortisol have been detected in more severe AD cases, which may be related to the progression of the disease. Increased cysteine associated with decreased uridine was the best‐paired combination to identify mild AD [25]. Overall, impaired amino acid metabolism is linked to the pathogenesis of AD, and altered amino acid signatures may serve as valuable biomarkers for diagnosing AD. Consequently, modulation of amino acid metabolism could represent a viable therapeutic strategy for AD.

While it has been hypothesized that cerebral glucose hypometabolism initiates the entire cascade of AD pathology [26], quantitative measurements of neuronal metabolic activity in STZ‐treated rats are limited. Our current MS/MS analysis study indicates that the ICV‐STZ model demonstrates neurometabolic impairments as commonly observed in AD. Additionally, to enhance our understanding of AD pathogenesis and assess the potential of novel therapeutic approaches, we used systems biology to evaluate the interactions between genes and metabolites and how they might contribute to disease progression.

## Materials and methods

### Establishment of AD model

#### Animal

**Fig. 1:**
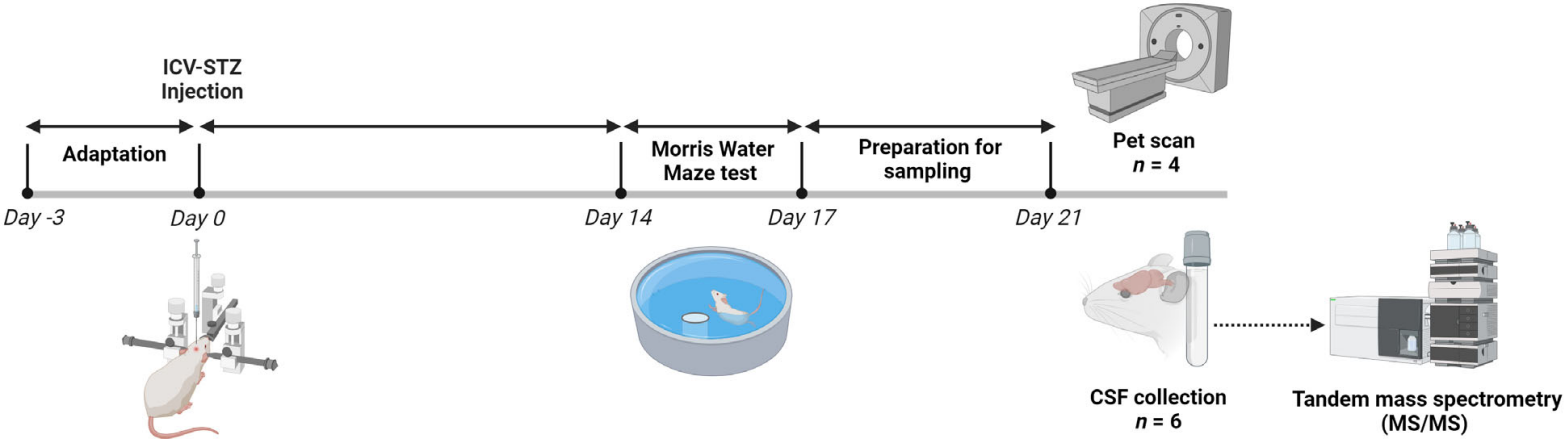
Schematic diagram of the experimental design.

Twenty male Wistar rats (200–220 g, 8 weeks of age) were housed in Plexiglas cages with woodchip bedding. The temperature was maintained at 22 ± 1 °C, with a light/dark cycle of 12/12 h. food and water were available *ad libitum*. All procedures involving animals and their care were conducted following the guidelines for the care and use of laboratory animals published by the National Institutes of Health (NIH No: 8023, revised 1978). Furthermore, this study was approved by the ethics and research Committee of the Tehran University of medical sciences (IR TUMS.NI.REC.1398.056). As a euthanasia method for rats, decapitation was used, and animal suffering was minimized by providing adequate housing, enrichment, and care to meet their natural needs. The experiments were conducted at 10 a.m. (Fig. 1).

#### Stereotaxic surgery

Rats were randomly assigned to two groups, sham‐ (controls) and STZ‐treated, with 10 rats in each group. STZ was dissolved in distilled water and administered based on a predetermined dosage for our rat model of AD [7, 27, 28]. Animals were anesthetized with ketamine (100 mg·kg^−1^) and xylazine (5 mg·kg^−1^) and mounted in a stereotaxic frame. A single ICV injection of STZ (3 mg·kg^−1^) was administered bilaterally, with 2.5 μL injected into each ventricle for 5 min on each side. The injection was carried out at a specific location in the brain: 0.8 mm posterior to the bregma, ±1.5 mm lateral to the sagittal suture, and 3.6 mm below the dura. This method was used to create a rat model of AD [29]. After injection, the needle was left in place for 3 min. The skin was sutured, and the animals were monitored before being returned to their cages. The sham group received a standard saline solution. Wound healing was monitored for 21 days before sampling. Two weeks after the STZ or saline injection, rats of both groups were divided into two categories: cognitive performance using the Morris Water Maze test (MWM, *n* = 6) and positron emission tomography (PET, *n* = 4) to confirm the AD‐like model and sampling.

#### Morris water maze test (MWM)

MWM tests were conducted following a 2‐week injection of STZ or saline. MWM was utilized to assess the cognitive function of rats, specifically in the areas of the hippocampus, to validate the previously established AD model [30]. MWM consists of a round pool with a radius of 110 cm and a depth of 70 cm, filled with tap water at 21 ± 1 °C. The maze is divided into four equal quadrants, and a hidden circular platform is located at one of the quadrants. The test comprises the acquisition phase (four trials daily for 3 days) and the probe test (one trial on 1 day). In the acquisition phase, animals were put in the water from different quadrants to find the hidden platform in each trial. If the animal failed to find the platform, it was guided to reach the platform and allowed to stay there for 30 s. The escape latency is recorded at 90 s. The time the rat spent on the platform (escape latency) was accounted for to assess the ability of spatial learning. The animals' swim paths were automatically recorded using a video camera‐based system (EthoVision, Noldus, Version 11, Wageningen, the Netherlands). The probe phase (for retention retrieval of memory) was performed 24 h after completion of the last acquisition phase session by removing the hidden platform and placing the animal into the water in one quadrant. The latency of the first entry to the platform location and the time spent in the target quadrant were analyzed (probe test).

#### Positron emission tomography (PET)

Following the MWM test, two groups of animals were scanned using an Xtrim PET microPET scanner. Rats were given a tail vein injection of 1 mCi of 18FDG under general anesthesia. Each small‐animal PET scan has three‐dimensional regions of interest (ROIs) carefully delineated around the brain. The brain‐to‐background ratio (BBR) was calculated from the ROIs as follows: (ROI counts per voxel)/(background counts per voxel) [31].

### CSF sample collection

CSF sampling was performed after 3‐weeks. Rats were put under ketamine anesthesia (100 mg·kg^−1^) and placed in a stereotaxic frame. The skull was stretched about 45°, revealing a depressible surface resembling a rhomb between the occipital protuberances and the atlas spine. A 23‐gauge needle was inserted into the cisterna magna to collect CSF. The PE‐50 Polyethylene tubing was inserted using the blunt end of the needle, and a collecting syringe was attached to the other end. Simple aspiration was used to withdraw the uncontaminated material into the syringe. To avoid contamination of blood, CSF samples (about 100 μL per rat) were centrifuged in 1500 ***g*** for 10 min at 4 °C, after which 25 μL was placed in a sterile filter paper for MS/MS analysis [32].

### Tandem mass spectrometry (MS/MS) analysis

Using isotope dilution mass spectrometry (MS), amino acids (in butyl‐ester forms) in CSF samples obtained from STZ and sham groups were assessed using a tandem mass spectrometry system (Shimadzu, CLAM2040, MS/MS, Kyoto, Japan) and commercial kits [CHROMSYSTEM MS/MS complete kits, amino acids in dried blood spots (DBS)], CHROMSYSTEM Co, Gräfelfing/Munich, Germany 10 μL of the sample was transferred to a microtube, and 200 μL of internal standard was pipetted on the sample; the microtube was recapped, vortexed for 10 s, and centrifuged (10 000 ***g***) at 4 °C for 10 min. Next, 150 μL of supernatant was transferred to a microplate, incubated for 20 min at 45 °C, and evaporated until completely dry. Subsequently, 50 μL of acetyl chloride 1‐butanol was added to the sample, followed by shaking for 1 min at 700 ***g***. Samples were incubated at 65 °C for 15 min and dried under nitrogen gas at 45 °C for 15 min, after which 50 μL of 75% acetonitrile was added. Finally, samples were shaken for 2 min and introduced to the MS/MS system. The injection volume was 10 μL, the flow rate was 150 μL·min^−1^, and an analysis time of 2.074 min was applied. MRM (multiple reaction monitoring) was adjusted as a scan mode [33, 34].

### Metabolite set enrichment analysis

Enrichment analysis was performed in CSF samples to investigate the biological concepts underlying changes in metabolites of interest (metabolanalyst 5, Edmonton, Alberta, Canada). Over‐representation analysis was conducted using two separate databases (KEGG and SMPDB). Next, the relative‐betweenness centrality (Rb‐C) and out‐degree centrality (Od‐C) parameters were considered during pathway analysis. The analysis of the outcomes obtained from the enrichment was conducted by evaluating the False Discovery Rate (FDR) < 0.05. Initially, the results were arranged according to their impact, and only the significant outcomes in terms of FDR were retained.

### Gene‐metabolite‐disease network reconstruction

To identify the genes associated with the significant metabolites of AD, we reconstructed a gene‐metabolite‐disease network using metaboanalyst 5 (MetaboAnalyst, Edmonton, Alberta, Canada). The outcomes derived from this network are presented in Table 4 and a Sankey diagram (https://sankeymatic.com/).

### Statistics

We used two‐way ANOVA with a Tukey's post‐test to statistically evaluate rats' performance during days 1 to 3 of the training period in the MWM test. Student's *t*‐tests were performed for the MWM probe test, changes in brain glucose metabolism in microPET images, and dilution mass spectrometry of amino acids in ICV‐STZ and control groups. Final data were analyzed using graph pad prism 8 (GraphPad Software, Inc. California, USA). Data were reported as mean ± SE, and *P* < 0.05 was considered statistically significant.

## Results

### Confirmation of AD‐like model

**Fig. 2:**
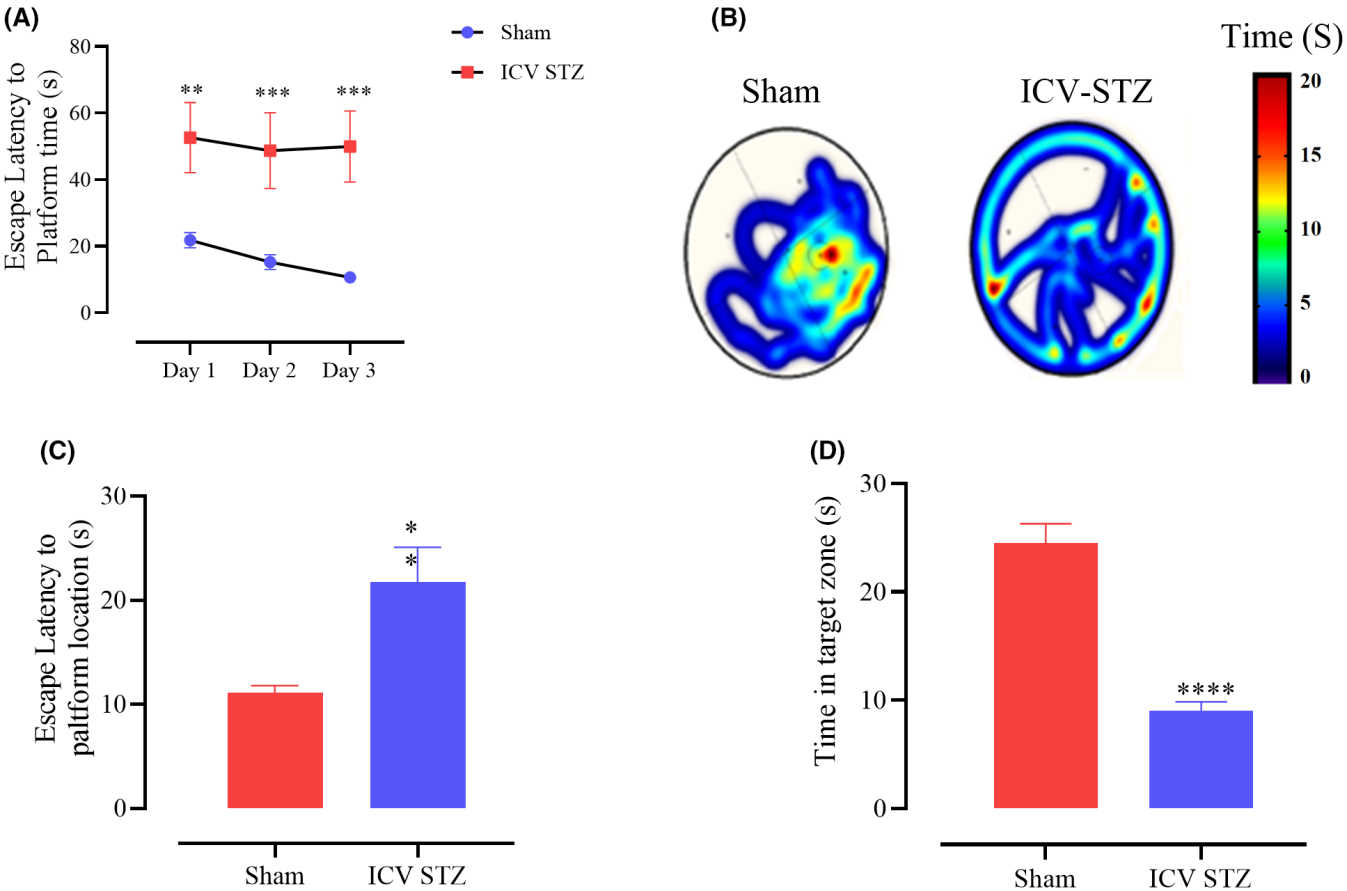
Assessment of learning and memory (Morris water maze test) in sham‐ and ICV‐STZ‐treated male rats. All animals were trained to find a hidden platform zone for 3 days. Then, a probe test was performed to determine the learning capacity of groups. (A) A Two‐way ANOVA of scape latency (the time to find the hidden platform) during the training phase in the ICV‐STZ compared to the sham group. (B) A comparison of the average heat map of MWM trials between groups. The heat map scale bar indicates the time in seconds (C) Student's *t*‐test analysis of scape latency to platform shows ICV‐STZ‐induced rats spent more time finding the hidden platform at the former location of the platform. (D) Student's *t*‐test analysis of time in the target zone shows ICV‐STZ‐induced rats spent less time in the target quadrant searching for the missing than did the sham group. (**P < 0.01, ***P < 0.001, and ****P < 0.0001 compared to the sham group; *n* = 10 rats in each group). Data are presented as mean ± SEM. ICV‐STZ, Intracerebroventricular‐streptozotocin.

Using the MWM test, we investigated the effects of ICV‐STZ on spatial learning and memory capacity compared to the sham group. Figure 2A shows the escape latency to locate the concealed platform during the acquisition phase of MWM. Based on two‐way ANOVA with a Tukey's post‐test analysis, sham‐ and STZ‐treated rats gradually improved performance from days 1 to 3 in the training period. The escape latency was noticeably and significantly lower in the sham group on all 3 days, demonstrating the detrimental effects of STZ on the learning process. A comparison of the heat maps between the two groups confirmed these results (Fig. 2B). Twenty‐four hours following the last training session, memory recall was evaluated using a probe trial test (Fig. 2C,D). The two criteria examined were the latency to initially reach the platform location and the duration spent in the target zone. Based on student *t*‐test analysis, first access to the platform location was significantly impacted by latency (*P* < 0.01), Fig. 2C, and the STZ group showed a stronger initial delay than the sham group upon arrival at the platform position. Furthermore, the time spent in the target quadrant significantly differed between the STZ and sham groups (*P* < 0.0001, Fig. 2D).

**Fig. 3:**
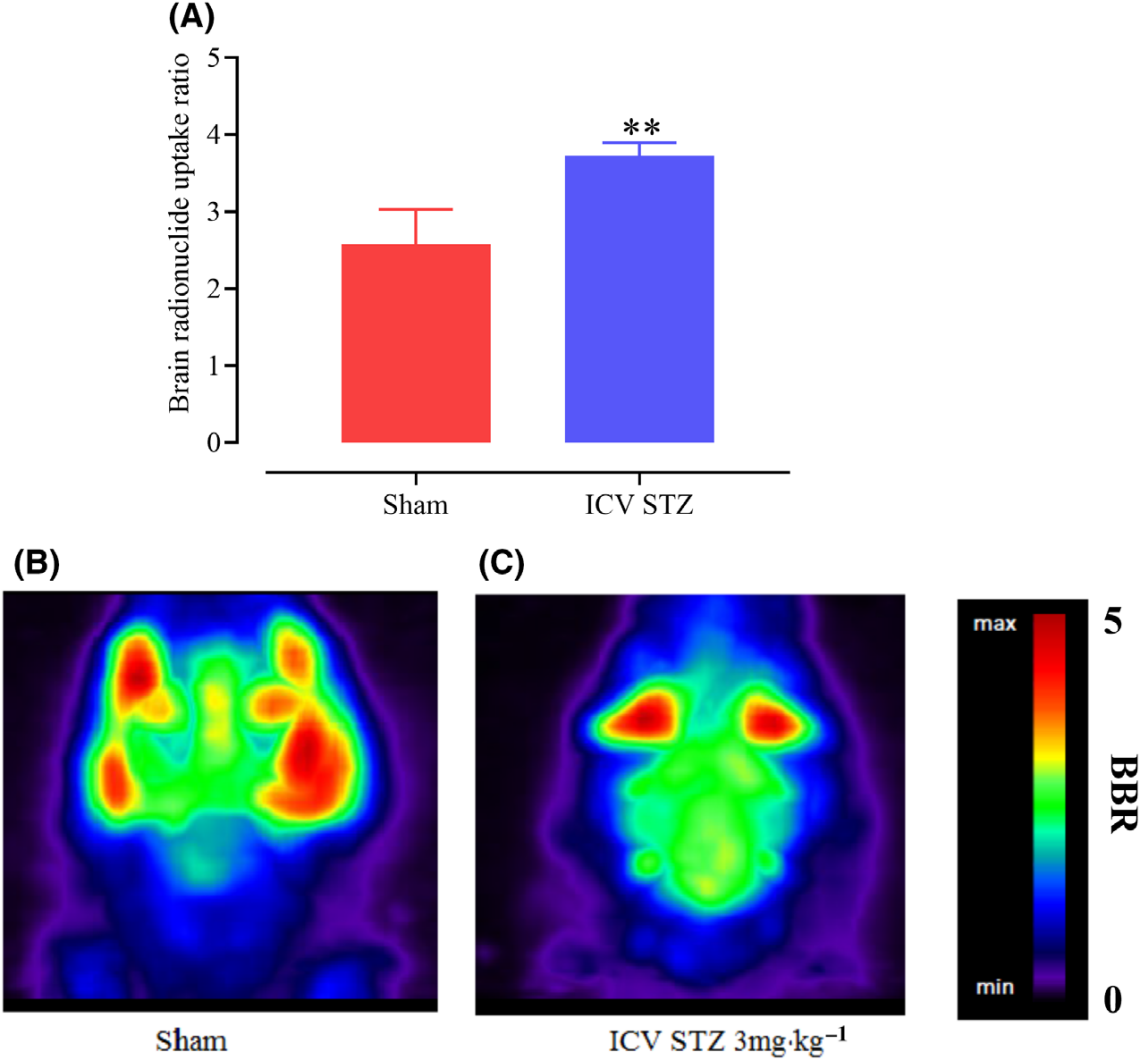
Changes in brain glucose metabolism in microPET images. (A) Student's *t*‐test analysis of the brain radionuclide uptake ratio value in the ICV‐STZ compared to the sham group. Rats were each injected via the tail vein with about 800 μCi of the ^18^FDG under general anesthesia. For each small‐animal PET scan, 3‐dimensional regions of interest (ROIs) were manually drawn around the brain, and the backgrounds were selected behind the backbone. The ROIs were converted to the brain‐to‐background ratio (BBR) as (ROI counts per voxel)/(background counts per voxel). (B, C) The amount of the radionuclide uptake ratio of the selected ROIs for 1 h PET scan of each rat. The small‐animal PET scans were obtained with a microPET scanner (Xtrim PET) at the Preclinical Core Facility (TPCF) based at Tehran University of Medical Sciences. (**P < 0.01 compared to sham group; *n* = 4 rats in each group.) Data are presented as mean ± SEM. BBR, Brain‐to‐background ratio; FDG, Fludeoxyglucose F18; PET, A positron emission tomography; ROIs, region of interest.

It has been shown that fluorodeoxyglucose (FDG) uptake stimulates a specific pentose phosphate pathway (PPP) triggered by hexose‐6P‐dehydrogenase (H6PD), which contributes to NADPH‐dependent antioxidant responses in the endoplasmic reticulum (ER) [35, 36]. Metabolic imaging using microPET in both AD patients and animal AD models revealed brain glucose hypometabolism as one of the most significant pathophysiological characteristics of the disease. We assessed 18FDG distribution in the rat brain by microPET and measured the BBR to evaluate brain glucose metabolism in ICV‐STZ and control animals. Our results indicated that FDG uptake was homogeneous in the hippocampus of sham controls, whereas it was selectively decreased in the brains of ICV‐STZ rats (*P* < 0.01). The change in brain glucose metabolism strongly suggests the development of glucose hypometabolism in brain tissue of ICV STZ animals (Fig. 3).

### Biochemical studies

Table 1. shows the dilution mass spectrometry (MS) analysis of amino acids in CSF samples from sham and ICV‐STZ animals. Our results indicate that the levels of a multitude of amino acids are significantly lower in the CSF of ICV‐STZ animals compared to controls (Fig. S1).

**Table 1:** The concentration of various amino acids in CFS samples obtained from sham‐ and ICV‐STZ‐treated rats. Tandem mass spectrometry was used to analyze all CFS samples for both groups. Based on student *t*‐test analysis, amino acids in CSF samples from sham and ICV‐STZ animals were compared.

Amino acid	Sham (μmol·L^−1^)	STZ (μmol·L^−1^)	*P* value	*t*, df
Alanine (Ala)*	7.550 ± 1.926	2.429 ± 1.120	0.0363	*t* = 2.384, df = 11
Arginine (Arg)*	3.255 ± 1.140	0.4151 ± 0.06791	0.0286	*t* = 2.487, df = 12
Citrulline (Cit)	8.184 ± 2.813	2.021 ± 1.503	0.0821	*t* = 1.932, df = 10
Aspartic acid (Asp)*	18.88 ± 6.930	3.737 ± 2.218	0.0480	*t* = 2.224, df = 11
Glutamic acid (Glu)*	3.000 ± 0.6226	1.026 ± 0.3500	0.0151	*t* = 2.873, df = 11
Glycine (Gly)*	2.527 ± 0.8037	0.8036 ± 0.1812	0.0455	*t* = 2.255, df = 11
Histidine (His)	1.846 ± 0.5103	0.9127 ± 0.3040	0.1422	*t* = 1.571, df = 12
Isoleucine (Ile)*	1.340 ± 0.1089	0.9705 ± 0.09087	0.0403	*t* = 2.606, df = 11
Leucine (Leu)	0.9616 ± 0.5379	0.2510 ± 0.1524	0.2278	*t* = 1.271, df = 12
Lysine (Lys)	1.312 ± 0.8715	0.05597 ± 0.01956	0.1752	*t* = 1.441, df = 12
Methionine (Met)**	1.495 ± 0.2329	0.3894 ± 0.1602	0.0021	*t* = 3.911, df = 12
Phenylalanine (Phe)***	1.610 ± 0.1831	0.4527 ± 0.1685	0.0006	*t* = 4.652, df = 12
Proline (Pro)*	2.439 ± 0.5223	0.7406 ± 0.4833	0.0381	*t* = 2.356, df = 11
Serine (Ser)*	3.865 ± 0.6944	1.357 ± 0.5329	0.0142	*t* = 2.910, df = 11
Threonine (Thr)*	4.258 ± 0.7227	1.735 ± 0.7934	0.0407	*t* = 2.318, df = 11
Tryptophan (Trp)*	0.2617 ± 0.03834	0.1236 ± 0.04558	0.0401	*t* = 2.281, df = 13
Tyrosine (Tyr)*	2.261 ± 0.4464	0.6689 ± 0.2970	0.0117	*t* = 2.969, df = 12
Valine (Val)*	3.859 ± 0.7953	1.679 ± 0.6335	0.0493	*t* = 2.168, df = 13

*P < 0.05, **P < 0.01, and ***P < 0.001 comparing sham versus ICV‐STZ. Data are presented as mean ± SEM (*n* = 7 rats in each group).

### Enrichment and pathway analysis results

**Fig. 4:**
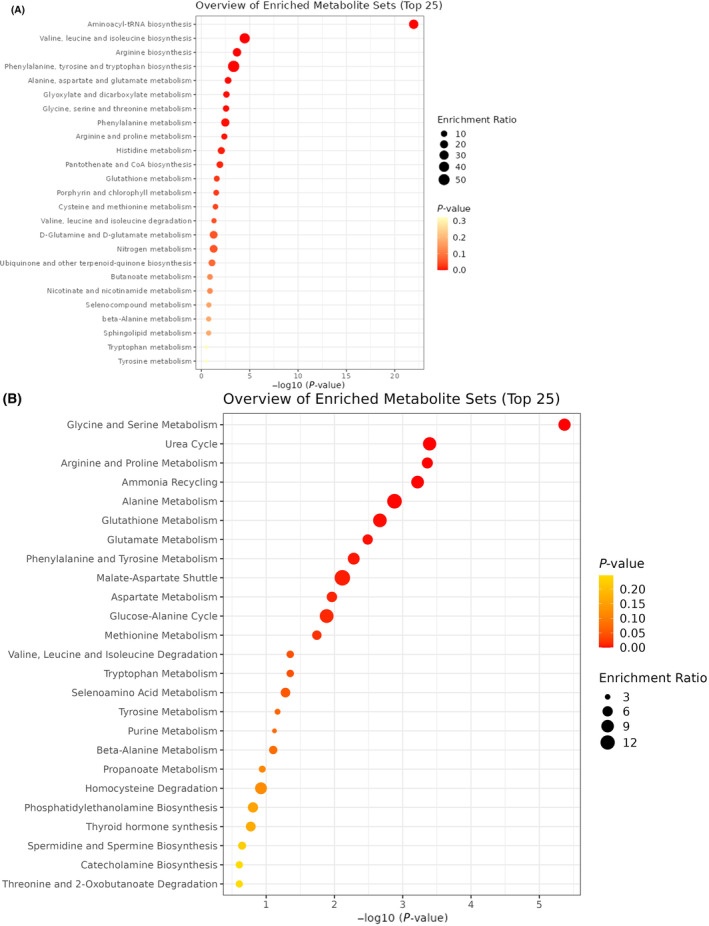
KEGG and SMPDB enrichment analysis. Results using the KEGG (A) and SMPDB (B) databases rendered 9 and 7 pathways, respectively. We used a FDR threshold of less than 0.05 to determine which pathways were significantly affected. FDR, False discovery rate.

Results of the enrichment and pathway analysis are depicted in Fig. 4 and Table 2. Nine pathways were identified via KEGG, 7 through SMPDB, 8 via Rb‐C, and 9 via Od‐C.

**Table 2:** Summary of KEGG and SMPDB enrichment analysis results. Outcomes were filtered based on a False Discovery Rate (FDR) threshold of less than 0.05. Moreover, two criteria were used to categorize the outcomes of the pathway analysis. The first criterion was based on the impact of the outcome, and the second was whether the outcome met the FDR threshold. Only results that met both criteria were retained.

Enrichment analysis				Pathway analysis			
KEGG	FDR	SMPDB	FDR	Rb – C		Od – C	
				FDR	Impact	FDR	Impact
Aminoacyl‐tRNA biosynthesis	9.21E‐21	Glycine and Serine Metabolism	0.000363	Phenylalanine, tyrosine, and tryptophan biosynthesis		Phenylalanine, tyrosine, and tryptophan biosynthesis	
				0.009971	1	0.0099708	0.75
Valine, leucine, and isoleucine biosynthesis	0.00138	Urea Cycle	0.0141	Glycine, serine and threonine metabolism		Aminoacyl‐tRNA biosynthesis	
				0.038556	0.50186	1.1821E‐20	0.51721
Arginine biosynthesis	0.0058	Arginine and Proline Metabolism	0.0141	Alanine, aspartate and glutamate metabolism		Glycine, serine and threonine metabolism	
				0.030546	0.42068	0.038556	0.42858
Phenylalanine, tyrosine, and tryptophan biosynthesis	0.00963	Ammonia Recycling	0.0155	Phenylalanine metabolism		Arginine biosynthesis	
				0.046716	0.35714	0.0061101	0.375
Alanine, aspartate and glutamate metabolism	0.029	Alanine Metabolism	0.0243	Arginine and proline metabolism		Alanine, aspartate and glutamate metabolism	
				0.046522	0.22166	0.030546	0.35484
Glyoxylate and dicarboxylate metabolism	0.0336	Glutathione Metabolism	0.0383	Arginine biosynthesis		Phenylalanine metabolism	
				0.0061101	0.19289	0.046716	0.3
Glycine, serine and threonine metabolism	0.0336	Glutamate Metabolism	0.0456	Aminoacyl‐tRNA biosynthesis		Valine, leucine and isoleucine biosynthesis	
				1.1821E‐20	0.16667	0.0014573	0.25
Phenylalanine metabolism	0.035			Glyoxylate and dicarboxylate metabolism		Glyoxylate and dicarboxylate metabolism	
				0.037698	0.14815	0.037698	0.1923
Arginine and proline metabolism	0.0393					Arginine and proline metabolism	
						0.046522	0.175

The final selection of affected pathways was based on whether they were detected by all four analyses (KEGG, SMPDB, Rb‐C, and Od‐C). The repeated pathways in three or four instances were chosen as the ultimate consequences (Table 3).

**Table 3:** The final selection of affected pathways was based on detection by all four types of analysis. Those items replicated three times were selected as final pathways.

	SMPDB	KEGG	Rb – C	Od – C	Result	Final decision
Aminoacyl‐tRNA biosynthesis	N	Y	Y	Y	3	+
Valine, leucine, and isoleucine biosynthesis	N	Y	N	Y	2	−
Arginine biosynthesis	N	Y	Y	Y	3	+
Phenylalanine, tyrosine, and tryptophan biosynthesis	N	Y	Y	Y	3	+
Alanine, aspartate, and glutamate metabolism	Y	Y	Y	Y	4	+
Glyoxylate and dicarboxylate metabolism	N	Y	Y	Y	3	+
Glycine, serine, and threonine metabolism	Y	Y	Y	Y	4	+
Phenylalanine metabolism	N	Y	Y	Y	3	+
Arginine and proline metabolism	Y	Y	Y	Y	4	+
Urea Cycle	Y	N	N	N	1	−
Ammonia Recycling	Y	N	N	N	1	−
Glutathione Metabolism	Y	N	N	N	1	−

### Gene‐metabolite‐disease network

**Fig. 5:**
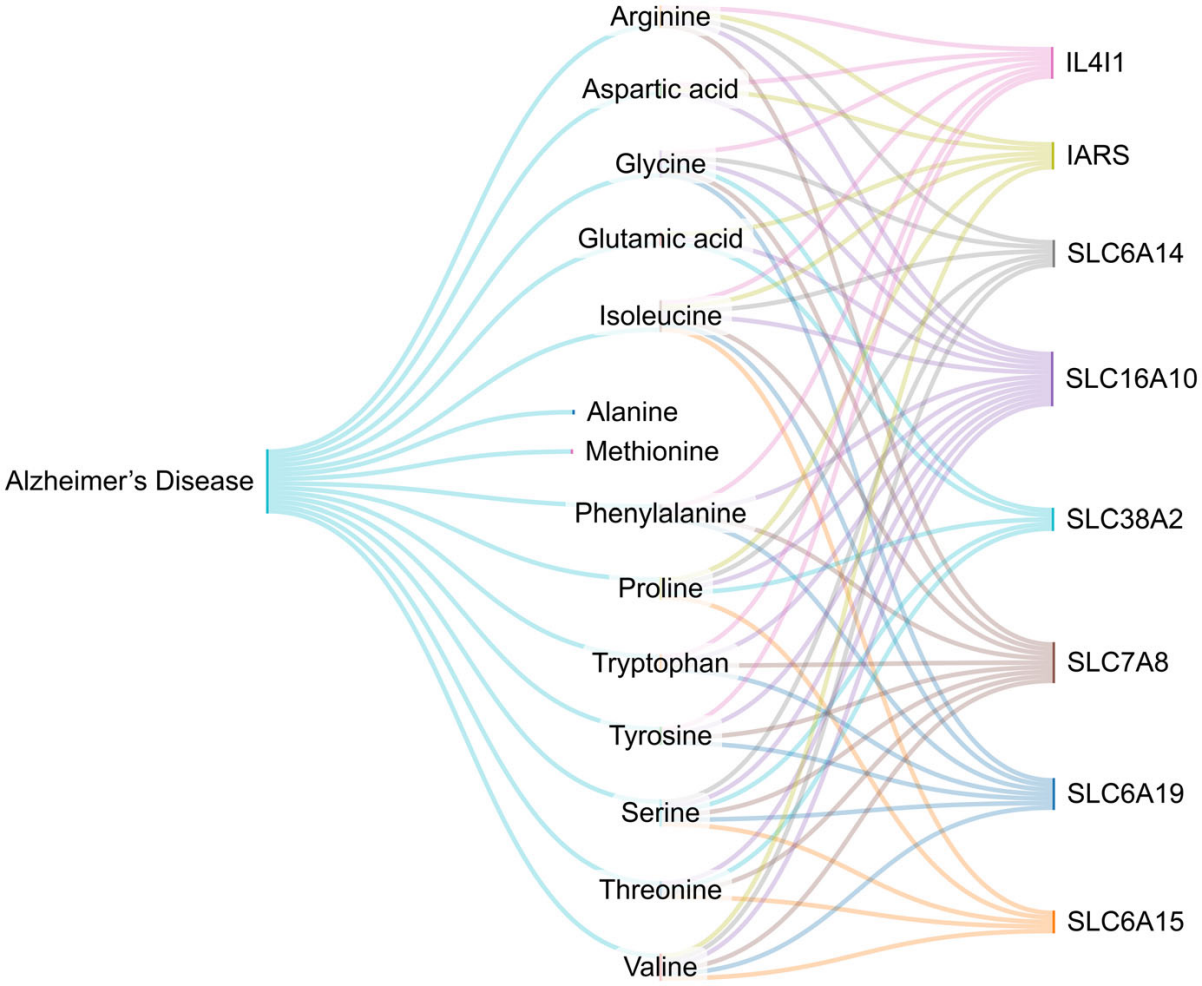
Diagram showing the relationship between genes and metabolites related to Alzheimer's disease. Eight genes (SLC16A10, SLC7A8, SLC6A19, IL4l1, SLC6A14, IARS, SLC38A2, and SLC6A15) are linked with significant metabolites that are pertinent to Alzheimer.

Generation of the gene‐metabolite‐disease network revealed that eight genes (SLC16A10, SLC7A8, SLC6A19, IL4l1, SLC6A14, IARS, SLC38A2, and SLC6A15) are linked to metabolites of which the CSF levels were significantly altered in our rat model of AD. The association between these genes and identified metabolites is outlined in Table 4 and Fig. 5.

**Table 4:** The association between the identified genes and metabolites. The amino acids isoleucine, glycine, serine, and valine are associated with more genes than other metabolites.

	Alzheimer's disease													
	Alanine	Arginine	Aspartic acid	Glutamic acid	Glycine	Isoleucine	Methionine	Phenylalanine	Proline	Serine	Threonine	Tryptophan	Tyrosine	Valine
SLC16A10	−	+	+	+	+	+	−	+	+	+	+	+	+	+
SLC7A8	−	+	−	−	+	+	−	+	−	+	+	+	+	+
SLC6A19	−	−	−	−	+	+	−	+	−	+	−	+	+	+
IL4I1	−	+	+	−	+	+	−	+	−	−	−	+	+	−
SLC6A14	−	+	−	−	+	+	−	−	+	+	−	−	−	+
IARS	−	+	+	+	−	+	−	−	+	−	−	−	−	+
SLC38A2	−	−	−	+	+	−	−	−	+	+	+	−	−	−
SLC6A15	−	−	−	−	−	+	−	−	+	+	+	−	−	+
	0	5	3	3	6	7	0	4	5	6	4	4	4	6

## Discussion

Globally, AD is one of the leading causes of dementia in older adults (≥ 65 years of age) [37]. The majority of research to date has focused on the AT(N) framework, the accumulation of beta‐amyloid (A) and phosphorylated tau (T) proteins, and the degeneration of neural structures (N) [38]. However, an emerging realization from that body of work and related approaches indicated that AD neurons are chronically malnourished. Thus, these neurons cannot effectively utilize the abundant nutrients and trophic factors available [39]. To provide new insights into disease etiology, we considered AD a metabolic disorder and employed metabolomics and systems biology.

According to our neurobehavioral findings from the MWM test, ICV‐STZ dramatically decreased learning and spatial memory performance. Multiple lines of research have explained how ICV‐STZ administration causes long‐term deficiencies in object recognition memory and spatial memory skills 3–4 weeks following treatment [30, 40, 41]. Additionally, our PET results revealed hypometabolism of glucose after administering ICV‐STZ. In AD patients, energy hypometabolism (particularly a decline in glucose metabolism) is one of the earliest and most common anomalies observed [42]. Insulin and insulin growth factor‐1 (IGF‐1) signaling are essential for maintaining metabolism and cognition in the CNS, and insulin resistance is one of the key risk factors for AD [43, 44, 45].

Our metabolomics study revealed a reduction in the concentration of various amino acids in the CSF of ICV‐STZ animals compared to sham controls. These amino acids can be categorized into the following families: branch‐chain amino acids (BCAAs; isoleucine, leucine, and valine), aromatic amino acids (phenylalanine, tyrosine, and tryptophane), charged amino acids (arginine, aspartic acid, and glutamic acid), sulfur‐containing amino acids (methionine), and others (alanine, glycine, proline, serine, and threonine). Consistent with our findings, another research demonstrated the alteration of CSF and plasma concentrations of 18 amino acids in Alzheimer's disease dementia (DAT). DAT cases had significantly lower plasma taurine and glutamate levels than controls. CSF glycine, leucine, and valine concentrations were reduced considerably in DAT cases. Additionally, the CSF to plasma (CSF/P) ratio for alanine, glutamine, glycine, phenylalanine, and valine was significantly lower in DAT cases than in controls [46]. Using NMR‐based metabolomics, 45 metabolites in CSF and 27 in serum were quantified in patients with mild cognitive impairment (MCI), AD, and controls. Results showed decreased valine levels in AD patients' CSF and increased valine degradation pathway metabolites (3‐hydroxyisobutyrate and α‐ketoisovalerate) [47]. According to the present study's findings and those of other researchers, using a quantitative metabolomics method may provide new understandings about the advancement of dementia and pinpoint significant changes in metabolic activity in CSF and blood serum.

BCAAs compete for entry into the brain with the aromatic amino acid phenylalanine. As a result, altering plasma BCAA levels may affect concentrations of crucial neurotransmitters in the brain, including serotonin, dopamine, epinephrine, and norepinephrine [48]. Research has demonstrated that limiting the consumption of BCAAs may benefit metabolic health. For example, in mice, a diet restricting BCAAs induced protective metabolic effects similar to a diet restricting protein [49]. Another study reported that increasing BCAA levels through dietary supplementation decreased neural growth factor (NGF) levels in the hippocampi of rats [50]. Interestingly, however, low levels of the BCAA family member leucine may benefit AD patients via modulation of mTOR signaling pathways [51]. An increase in amyloid‐beta levels results in augmented mTOR activation, which increases protein translation, leading to an increase in tau levels, a significant component of AD neurofibrillary tangles [52]. These observations indicate that BCAAs, particularly excessive levels, could impact AD progression.

As AD progresses, aromatic amino acids play a significant role. Tryptophan, a vital amino acid in the brain, can undergo two distinct metabolic fates in the brain: it can either be converted to serotonin or metabolized to α‐amino‐β‐carboxymuconate‐ε‐semialdehyde (ACMS) through the kynurenine pathway (KP) [53, 54]. It was indicated that oral tyrosine administration improves memory and cognitive function in the AD brain [55]. Furthermore, dysregulation of phenylalanine metabolism in the human hippocampus may contribute to the development of AD pathology [56]. Thus, (metabolic) dysregulation of aromatic amino acids can be linked to AD pathology.

Aspartate and glutamate are acidic amino acids, while arginine, lysine, and histidine are classified as basic amino acids. These amino acids all appear to decrease in the brains and plasma of AD patients. In addition, glutamate, histidine, and aspartate concentrations were also decreased in AD serum. Accumulating evidence suggests that aspartate and glutamate levels are reduced in the temporal lobes of the cerebral cortex of AD patients [14, 57, 58]. Autopsy brain specimens from AD patients exhibited decreased lysine and aspartate, while glutamate levels were increased [59]. In a mouse model of AD, Chen and colleagues demonstrated that glutamine supplementation reduced tau phosphorylation and inflammation [60]. When glucose metabolism is disrupted in neurodegenerative diseases such as AD, glutamine, glutamate, aspartate, alanine, and purines are likely to be degraded to serve as alternative energy sources [61]. Alanine, aspartate, and glutamate are readily metabolized in the brain because high levels of aminotransferases are present for the first step of their breakdown [57, 62].

Our study shows that glycine, proline, serine, and threonine are also significantly reduced in the CSF of ICV‐STZ‐ compared to sham‐treated rats. Glycine, the smallest amino acid, confers neuroprotection against D‐galactose‐induced neurodegeneration and memory impairment in the mouse brain. Accordingly, it was suggested glycine may be an effective neurotherapeutic candidate for treating age‐related neurodegenerative disorders [63]. Khalaji *et al*. [64] demonstrated that a proline‐rich peptide (PRP‐1) protected against AD‐related alterations induced by amyloid peptides in a rat model. D‐serine plays a significant role in cognitive impairment associated with normal aging and dementia [65]. This is further supported by observations indicating that increased brain and CSF d‐serine levels are associated with AD [66]. Another study reported significantly lower threonine CSF concentrations in AD patients compared to the control groups [67]. Methionine increases Aβ and phosphorylated tau levels in the brain. A decrease in methionine intake has been reported as a nutritional intervention to reduce Alzheimer's‐like neurodegeneration in mice by reducing the levels of Aβ formed [68]. Undoubtedly, imbalance or malnutrition of amino acids (alanine, arginine, aspartic acid, glutamic acid, glycine, isoleucine, methionine, phenylalanine, proline, serine, threonine, tryptophane, tyrosine, and valine) may accelerate the progression of AD.

Our gene‐metabolite‐disease network analysis revealed that eight genes (SLC16A10, SLC7A8, SLC6A19, IL4l1, SLC6A14, IARS, SLC38A2, and SLC6A15) are linked to metabolites of which the CSF levels were significantly altered in our rat model of AD. A major subgroup of membrane proteins, the solute carriers (SLCs) proteins, are responsible for transporting essential substances such as sugars, amino acids, nucleotides, inorganic ions, lipids, and drugs over the cell membrane into the cell [69]. These proteins are crucial to neuronal signaling during energy production in healthy neurons [70]. SLC6 belongs to the sodium‐ and chloride‐dependent neurotransmitter transporter family and is responsible for transporting gamma‐aminobutyric acid (GABA), glycine, proline, leucine, alanine, dopamine, serotonin, creatine, glutamate, and other substrates, thereby contributing to the regulation of neurotransmitter levels as part of CNS homeostasis [71]. Several subtypes of the SLC7 cationic amino acid transporter/glycoprotein‐associated family are expressed in the brain and can transport neutral and cationic amino acids. Notably, they influence the distribution of L‐DOPA, an amine or amino acid‐structured neurotransmitter precursor that plays a critical role in diseases such as schizophrenia and Parkinson's disease (PD) [72, 73]. Additionally, SLC16, a monocarboxylate transporter family member, can transport lactate, pyruvate, ketone bodies, and monocarboxylates. SLC38, belonging to the sodium‐coupled neutral amino acid transporter family, can transport neutral amino acids [74].

Neuroinflammation constitutes an essential element of AD and occurs when the brain's immune system is activated due to the buildup of aberrant proteins, including amyloid‐beta plaques and tau tangles. The presence of this inflammatory response may exacerbate neuronal injury and further deteriorate cognitive impairment [75]. Our results indicated that the IL4l1 gene was linked to the changes in metabolic profile observed in CSF of STZ‐treated rats. Interleukin (IL)‐4 is a key cytokine that regulates the alternative activation of macrophages. In tissue macrophages, including microglia, IL‐4 contributes to the diminution of pathological inflammation and the expression of anti‐inflammatory molecules such as arginase‐1, IL‐10, and transforming growth factor (TGF)‐β, which aid tissue repair [76]. IL‐4 injections into the hippocampal cavity of APP/PS1 mice increased arginase‐1‐expressing microglia and decreased Aβ plaque density 5 days after injection [77]. Furthermore, a decrease in gliosis and a reduction in neuron loss were associated with this change in cytokine levels [78]. In a 3xTg mouse model of AD, acute intracranial IL‐4 was also shown to have some positive effects [79]. Based on these studies, IL‐4 may have a neuroprotective effect on parts of the hippocampus that are more susceptible to aging and neurodegeneration [80].

The brains of individuals with AD and animal models have heightened aberrant neuronal metabolism, which results in cognitive impairment and many problems. Recent studies have emphasized the connection between AD and systemic metabolic alterations, including reduced glucose and oxygen metabolism, abnormal lipid metabolism (lipid peroxidation), disrupted Aβ metabolism and transport, and imbalances in biogenic metallic elements [81]. Researchers have recently suggested using the name ‘type‐3‐diabetes’ to refer to AD due to the similar molecular and cellular characteristics it has with type‐1‐diabetes, type‐2‐diabetes, and insulin resistance. These similarities are connected with older adults' memory impairments and cognitive decline [82]. Researchers demonstrated that insulin deficiency exacerbates cerebral amyloidosis and behavioral deficits in an insulin‐deficient diabetic AD transgenic mouse model induced by STZ [83]. The study's findings indicate that C57BL6/J mice treated with ICV‐STZ have neurometabolic abnormalities that are often reported in AD. In STZ‐treated mice, the levels of creatine, GABA, glutamate, and NAA were decreased, while the level of myo‐inositol was elevated. There was a notable decrease in the labeling of aspartate‐C3, glutamate‐C4/C3, GABA‐C2, and glutamine‐C4 from [1,6‐^13^C_2_] glucose. Consequently, the rate at which glucose is oxidized in the cerebral cortex and hippocampus of mice treated with STZ is reduced. This was caused by glucose oxidation decreases in glutamatergic and GABAergic neurons [84]. Since glucose hypometabolism is one of the early and persistent signs of Alzheimer's disease, along with the fact that Alzheimer's brains possess impaired insulin signaling, icv STZ injections are used by some researchers to test pharmacological therapies for AD in preclinical settings [85, 86]. It has, however, been found that the long‐term (120‐days) effects of ICV‐STZ do not appear to compromise the cholinergic system, so this model may not be the most efficient model for studying AD's cholinergic system or may only be informative for a short time (30‐days) [87]. Although the STZ model is valuable for investigating Alzheimer's disease metabolism, it does include some constraints. (a) The STZ model incompletely reproduces all the characteristics of Alzheimer's disease seen in people. (b) It primarily concentrates on certain parts of the condition, perhaps overlooking other significant components. (c) STZ‐induced damage manifests quickly, in contrast to the slow development of Alzheimer's disease in people.

One of the strengths of this work was its successful assessment of the amino acid profile in CSF samples obtained from male rats with sporadic Alzheimer's‐like disease induced by ICV‐STZ administration. Furthermore, using the gene‐metabolite‐disease network, we found that specific genes were associated with significant changes in amino acid profile to contribute to AD progression. The current study, however, had some limitations. One of the most critical limitations of the study was the lack of measuring other AD biomarkers in CSF, such as tau protein [88], taurine [89], beta‐amyloid, and beta‐amyloid/tau, as well as their potential impact on amino acid concentrations. Measuring the level of alteration of amino acids in CSF/plasma poses another limitation. In addition, the exact mechanisms underlying STZ toxicity in the brain remain largely unexplained and need further research. Hence, it is essential to acknowledge that the animal model used in this study may not accurately represent the fundamental pathophysiological mechanisms seen in most AD patients. Consequently, caution should be exercised when extrapolating the findings of this study. Future in‐depth molecular investigations are warranted to confirm the link between specific gene expression and changes in CSF amino acid profile.

## Conclusion and future perspective

In conclusion, our results revealed a reduction in the concentration of various amino acids in the CSF of ICV‐STZ‐ compared to sham‐treated rats. Moreover, we identified several genes associated with the altered amino acid profile via a gene‐metabolite network. Learning and spatial memory deficits were observed 2 weeks after injection with the STZ solution. As part of our study, we measured amino acid levels in CSF 3 weeks after STZ injection. Overall, this study demonstrates amino acid CSF levels are altered by inducing AD‐like symptoms in our rat model, changes which are likely associated with specific genes regulating transmembrane transport and cytokine function. These findings might facilitate the development of future therapeutic (nutritional and/or pharmacological) strategies aimed at preventing and/or restoring potential metabolic dysregulations in AD patients to interfere with disease progression or alleviate some of its symptoms.

## Conflict of interest

The authors declare no conflict of interest.

## Author contributions

ABB contributed to methodology, investigation, data curation, writing—original draft, writing—review & editing, and visualization. HL‐N contributed to methodology, investigation, data curation, writing—original draft, writing—review & editing, and visualization. JL contributed to methodology, investigation, and data curation. FK contributed to conceptualization and review & editing. SS contributed to the validation and review & editing. SG contributed to validation and writing—review & editing & supervision. JFB contributed to conceptualization, methodology, resources, writing—review & editing, supervision, project administration, and funding acquisition.

## Supporting information


**Fig. S1.** Amino acid chromatogram in various samples.

## Data Availability

The data supporting this study's findings are available from the corresponding author, Javad Fahanik Babaei, upon reasonable request.
