# Supplementary figures and images for: CSF amino acid profiles in ICV‐streptozotocin‐induced sporadic Alzheimer's disease in male Wistar rat: a metabolomics and systems biology perspective
Source: FEBS Open Bio. 2024 May 20;14(7):1116–32. doi: 10.1002/2211-5463.13814 (PMC11216934; doi:10.1002/2211-5463.13814)

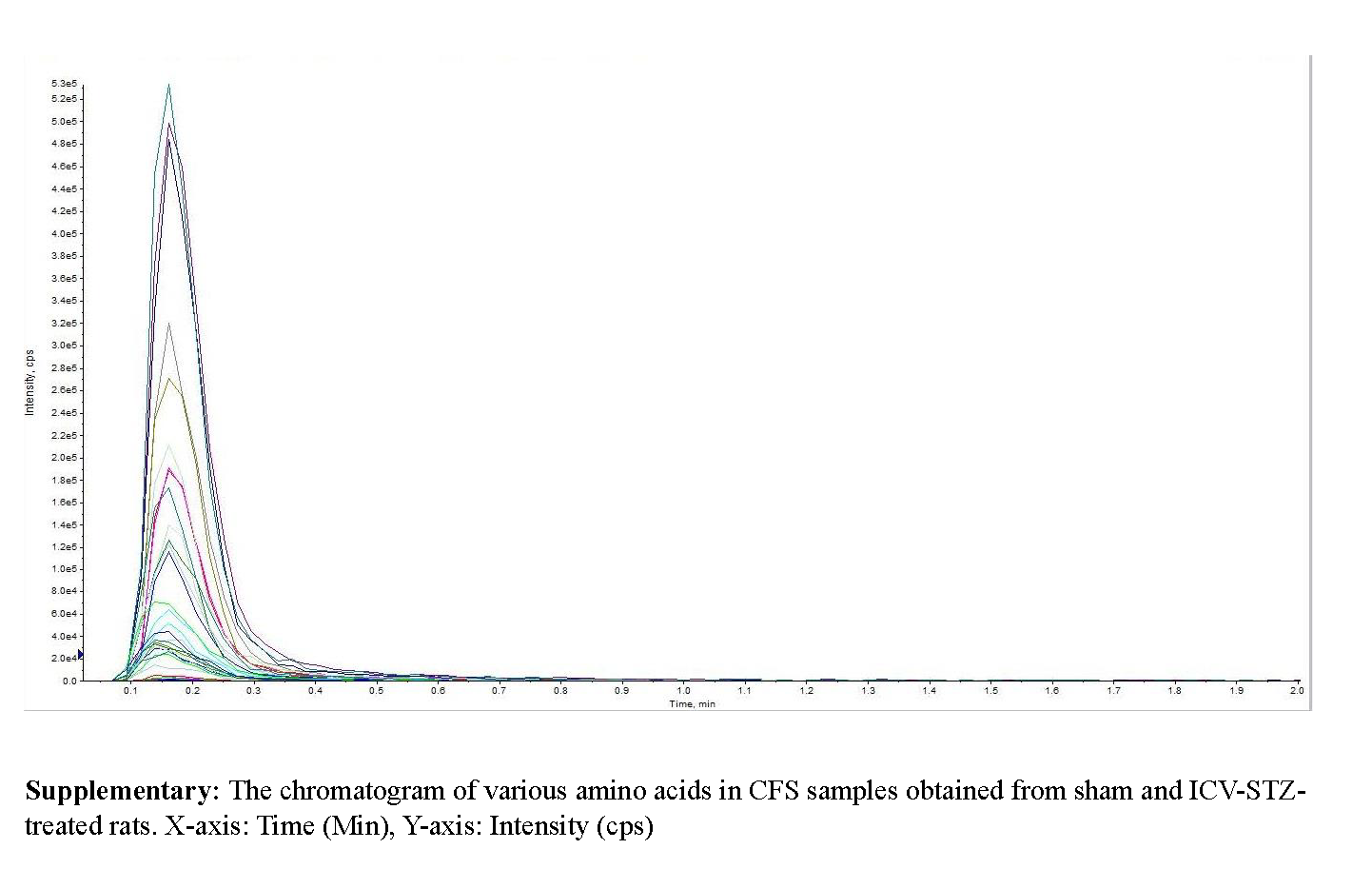

Supplement: Supplementary file 1 — Fig. S1. Amino acid chromatogram in various samples. [file FEB4-14-1116-s001.tif]
